# Editorial: Strategies in overcoming the chemoresistance in colorectal cancer

**DOI:** 10.3389/fonc.2023.1295204

**Published:** 2023-10-02

**Authors:** Sona Vodenkova, Tomas Buchler, Veronika Vymetalkova

**Affiliations:** ^1^ Department of Molecular Biology of Cancer, Institute of Experimental Medicine of the Czech Academy of Sciences, Prague, Czechia; ^2^ Biomedical Centre, Faculty of Medicine in Pilsen, Charles University, Pilsen, Czechia; ^3^ Department of Oncology, Second Faculty of Medicine, Charles University and Motol University Hospital, Prague, Czechia; ^4^ Institute of Biology and Medical Genetics, First Faculty of Medicine, Charles University, Prague, Czechia

**Keywords:** chemoresistance, colorectal cancer, platinum derivates, traditional Chinese medicine, fruquintinib, radiotherapy, adjuvant chemotherapy

Colorectal cancer (CRC) may display innate or acquired chemoresistance, and each is important in determining initial and subsequent lines of systemic treatment. Innate resistance is noted during early therapy phases, while acquired resistance develops during systemic antineoplastic treatment (1).

To prevent ineffective treatment and to select optimal regimens, there is an urgent need to identify biomarkers associated with therapy response. Therefore, the identification and validation of biomarkers for prediction and monitoring of the response of CRC patients to a specific regimen might shift the therapy towards precision medicine.

In this Research Topic, entitled “*Strategies in Overcoming the Chemoresistance in Colorectal Cancer*”, in a collection of ten papers the authors focused on the different aspects that play significant roles in the therapy efficacy. These studies provided insights into the potential of new therapeutic strategies (Zhang et al., Zheng et al., Hu et al., Jiang et al., Lin et al.), presented new predictive markers (Li et al.) or different prospectives and possibilities of applying adjuvant therapy in rectal cancer patients (Kuo et al.) as well as molecular mechanisms involved in the treatment response of CRC patients (Horak et al., Moretton et al., Liu et al.).

## New therapeutic strategies

Many natural compounds that have been used in Eastern medicine for centuries are currently being reevaluated in the context of current treatment approaches. Many of them are well tolerated by the patients and do not display toxic effects even at high doses (2). Their interactions with conventional chemotherapeutics represent a new direction in cancer therapy research. Lin et al. explored the potential of traditional Chinese medicine (TCM) as a sensitizer of anti-CRC drugs, and they believe TCM has a promising future as a natural, less toxic, alternative, and complementary therapy for clinical CRC treatment in developing new sensitizers to other anticancer drugs.

Jiang et al. focused on signalling targets controlled by protein tyrosine kinases to emphasize the potential of kinase inhibitors as treatment agents for metastatic CRC (mCRC). Over 50 tyrosine kinase inhibitors (TKIs) have been approved by the U.S. Food and Drug Administration (3). However, around 42 TKIs demonstrating preclinical antitumour activity, and despite numerous clinical trials, only regorafenib has been approved for clinical use in mCRC.

Zhang et al. studied fruquintinib, a small-molecule TKI that targets the vascular endothelial growth factor receptor. The authors focused on a combination of fruquintinib with PD-1 inhibitors in patients with microsatellite stable (MSS) or mismatch repair-proficient (pMMR) advanced CRC. They discovered that this combination evinced antitumor activity and manageable safety in MSS/pMMR advanced CRC patients.

Similarly, Hu et al. analysed the effect of Sorafenib, an oral multi-kinase inhibitor with a tumour-suppressing effect targeting the RAF-MEK-ERK pathway. However, its clinical application is limited due to complex drug resistance and many side effects. GW5074, one of the C-RAF inhibitors, has the potential to enhance chemotherapy efficiency. The authors demonstrated that GW5074 served as a Sorafenib sensitizer through mitochondria dysfunction and thus might reduce the risk of chemoresistance in CRC.

In a study by Zheng et al., a potentially targetable surface molecule in cancer cells, protocadherin 7 (PCDH7) was studied. One of the described and identified properties of PCDH7 was found to facilitate the development of chemoresistance in CRC cells by positively modulating Mcl-1 expression.

## Radiotherapy resistance in rectal cancer patients and treatment planning

Li et al. focused on the complication of lipoprotein receptor-related protein-1 (LRP-1) and survivin-associated radiotherapy resistance. They demonstrated that radiomics analysis of dynamic contrast-enhanced magnetic resonance imagining promotes preoperative assessment of LRP-1 and survivin expression in locally advanced rectal cancer and that their model displays significant potential to diagnose patients with radiotherapy resistance.

## Different prospectives and possibilities of applying adjuvant therapy in rectal cancer patients

Kuo et al. studied whether to administer adjuvant therapy for patients with rectal cancer with good response (ypT0-2N0) after neoadjuvant chemoradiotherapy and surgery. Based on the outcomes from 720 patients, the authors stated that in these circumstances adjuvant chemotherapy can be omitted.

## Functional studies

DNA damaging agents are frequently used in different therapy regimens and DNA damage response pathways are involved in the mechanism of chemoresistance (Horak et al.). Platinum derivatives, inducing highly cytotoxic DNA crosslinks, are one of the keystone chemotherapeutics in CRC. Since cisplatin treatment is often associated with high toxicity and, eventually, resistance, scientists are trying to develop more active and less toxic derivatives. Moretton et al. synthesized a series of clickable cisplatin derivates as a molecular tool that can be used for the identification, visualization, localization, and isolation of DNA-cisplatin crosslinks, and thus understanding the chemoresistance mechanisms.

Oxaliplatin is used to treat mCRC (1). The innate or acquired resistance to oxaliplatin-based combinations is still the leading cause of treatment failure. Horak et al. identified that microRNA miR-140 led to MRE11 downregulation and improved oxaliplatin therapy response in CRC.

With the outset of targeted therapy, the survival of CRC patients has substantially improved. Over the last two decades, targeted therapy for CRC has made considerable progress and is used in a majority of patients with mCRC throughout their treatment. However, resistance to targeted therapy also occurs and represents a significant clinical problem. Thus, investigating the resistance mechanism and finding strategies to overcome the resistance to targeted therapy is a constant challenge in the treatment of mCRC patients and is also a Research Topic of study of Liu et al.. The authors studied platycodon-D, a bioactive compound isolated from the Chinese herb platycodon grandiflorum, to inhibit PI3K/Akt pathway during the treatment of cetuximab in *KRAS*-mutated CRC cells. In this functional study, the increased sensitivity of *KRAS*-mutated CRC cells to cetuximab after platycodon-D treatment was recorded.

In summary, this Research Topic illustrates the several aspects of overcoming or preventing the failure of cancer therapy. We are aware we did not manage to cover all aspects of the issue of chemoresistance, however, we believe this Research Topic has shed light on many elements and will be helpful for further research on the issue of chemoresistance in CRC patients.

## Author contributions

SV: Conceptualization, Funding acquisition, Writing – original draft, Writing – review & editing. TB: Supervision, Writing – review & editing. VV: Conceptualization, Funding acquisition, Supervision, Writing – original draft, Writing – review & editing.
